# Supplementary figures and images for: Field evaluation of the performance of seven Antigen Rapid diagnostic tests for the diagnosis of SARs-CoV-2 virus infection in Uganda
Source: PLoS One. 2022 May 10;17(5):e0265334. doi: 10.1371/journal.pone.0265334 (PMC9089886; doi:10.1371/journal.pone.0265334)

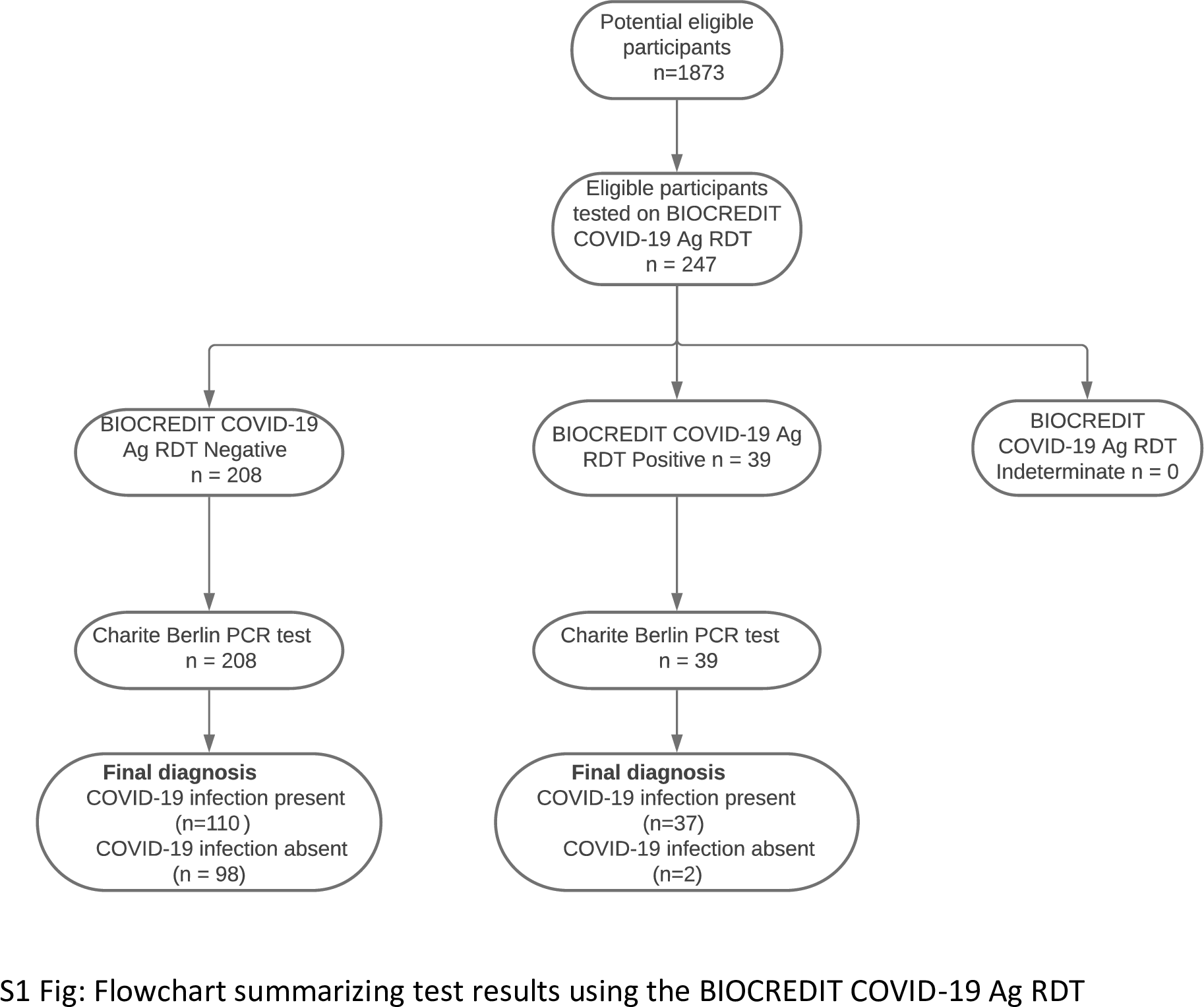

Supplement: S1 Fig — (TIF) [file pone.0265334.s001.tif]

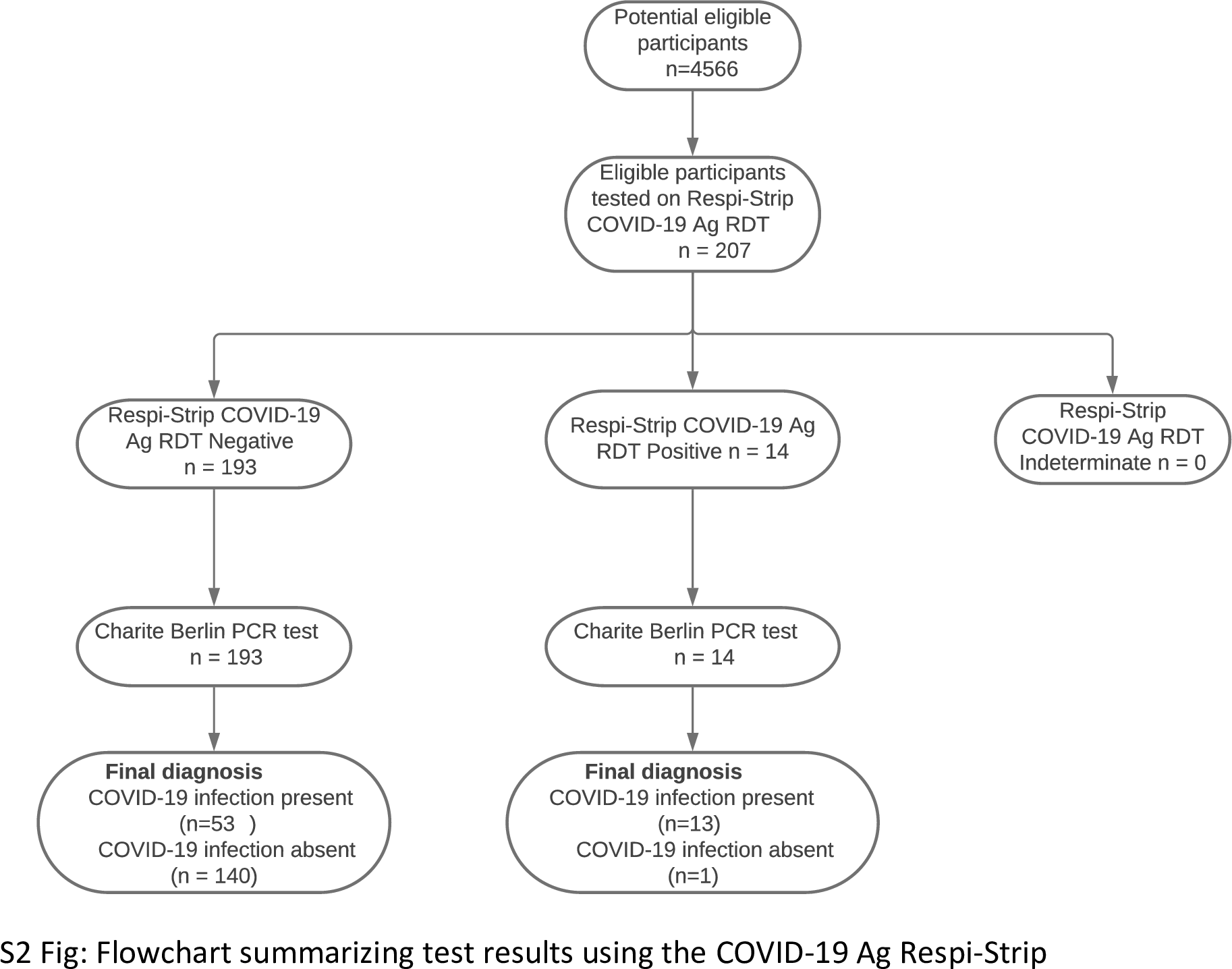

Supplement: S2 Fig — (TIF) [file pone.0265334.s002.tif]

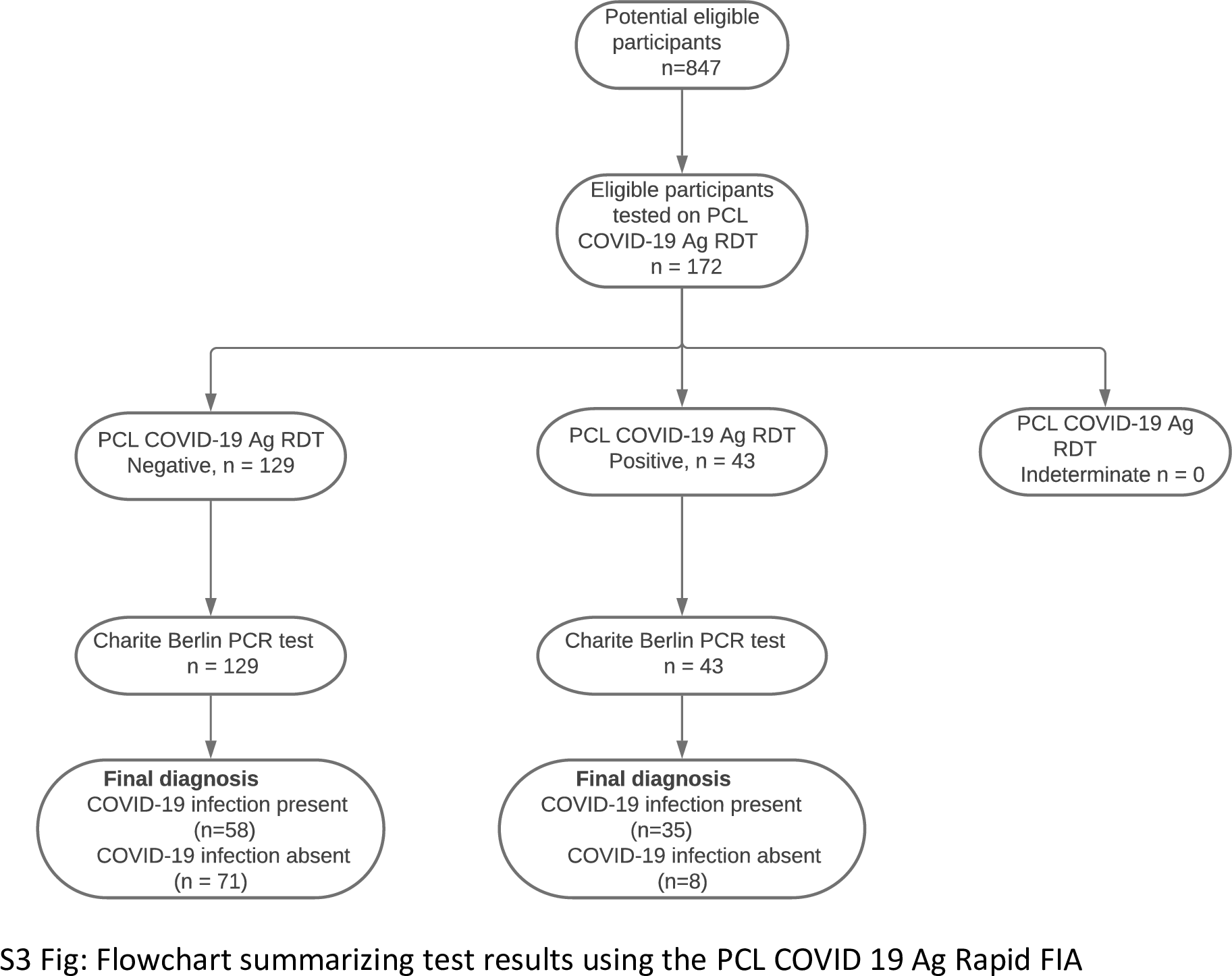

Supplement: S3 Fig — (TIF) [file pone.0265334.s003.tif]

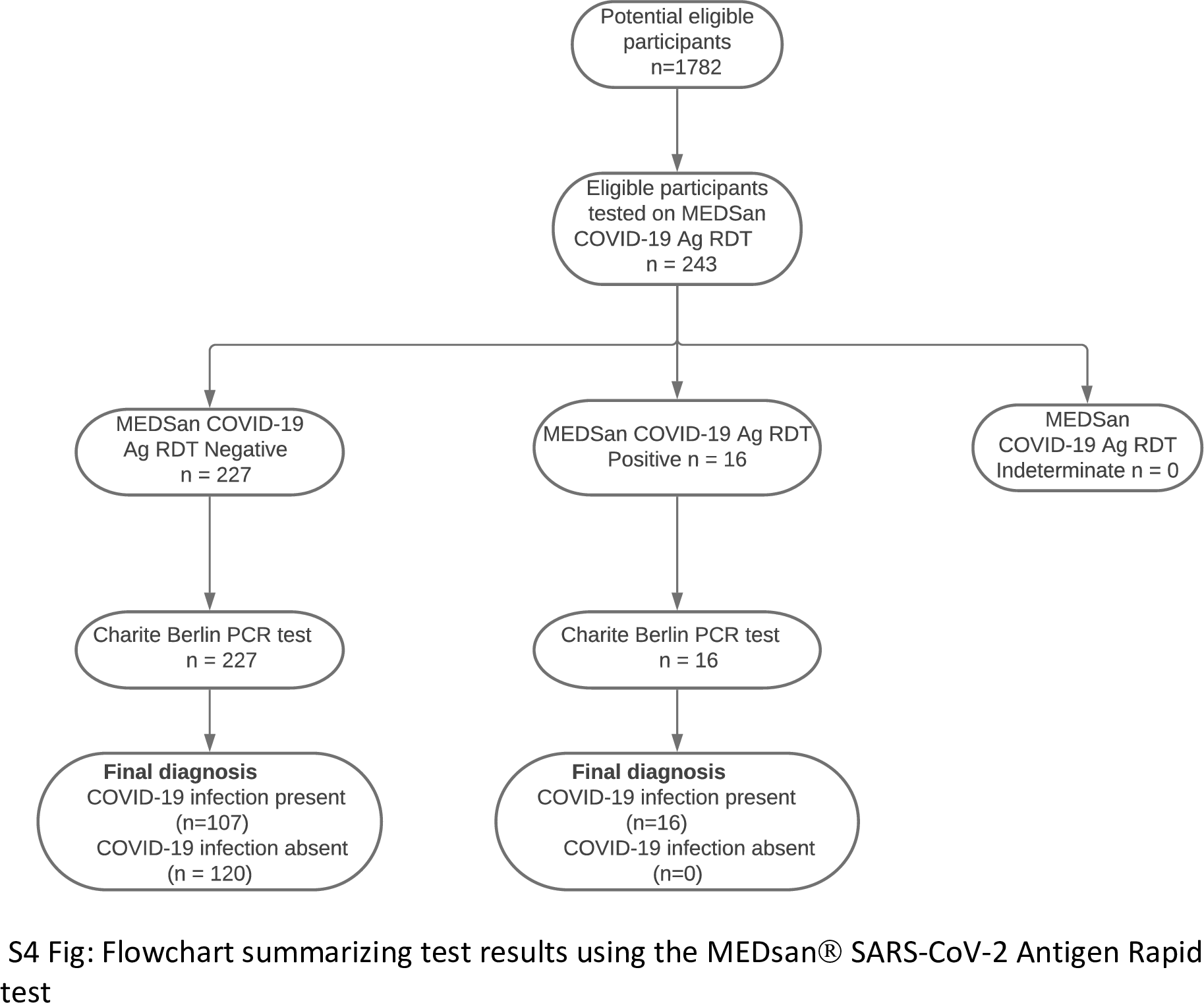

Supplement: S4 Fig — (TIF) [file pone.0265334.s004.tif]

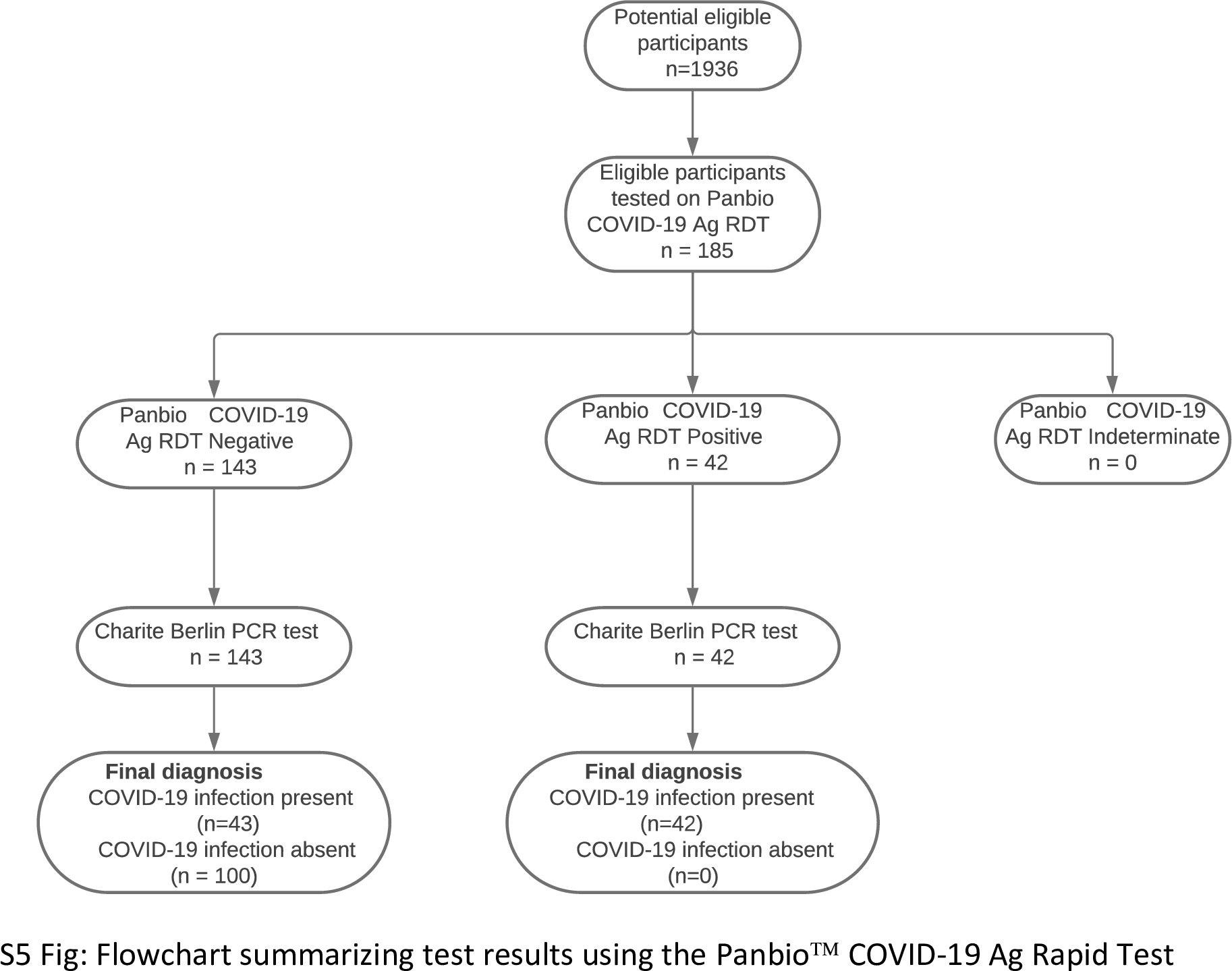

Supplement: S5 Fig — (TIF) [file pone.0265334.s005.tif]

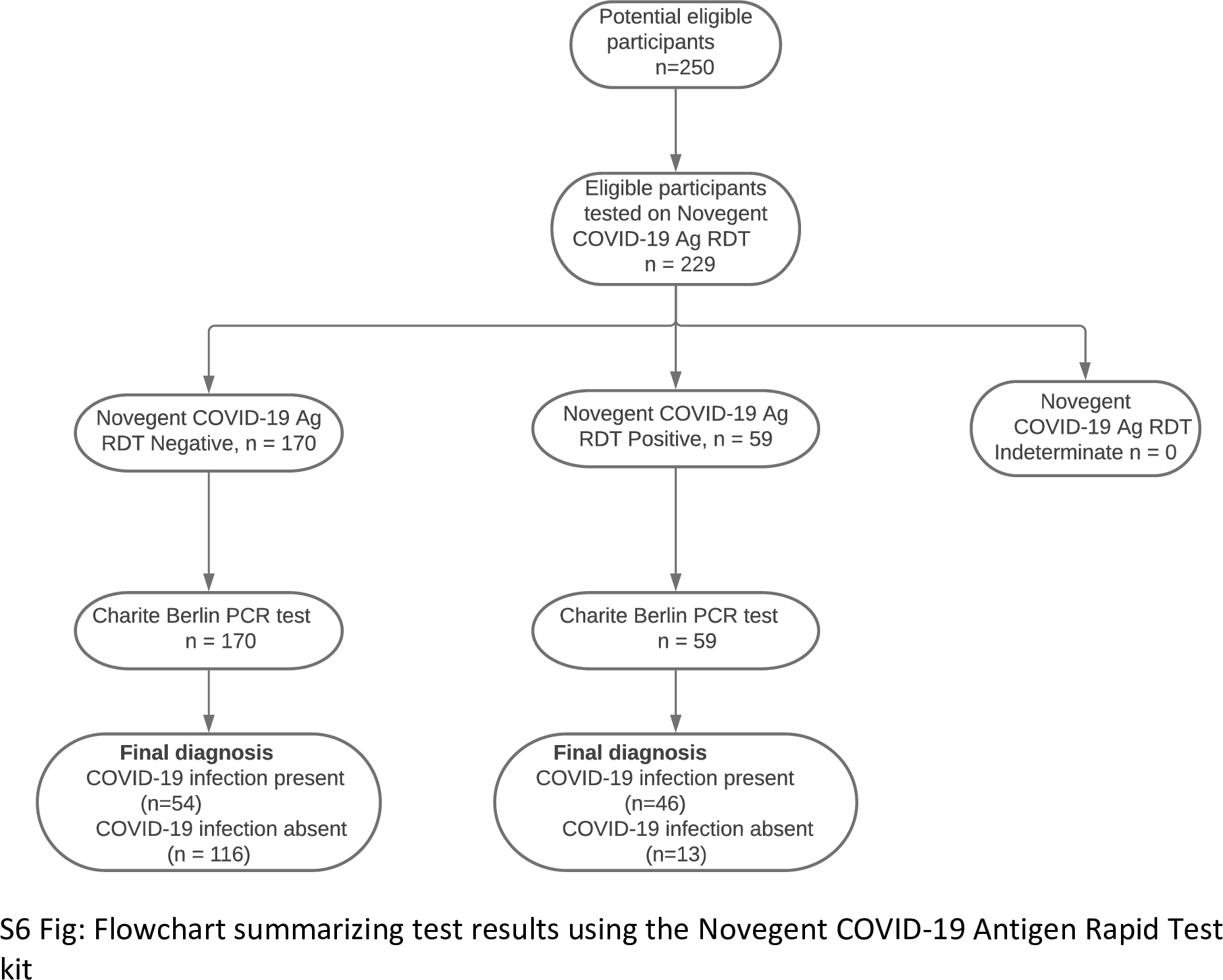

Supplement: S6 Fig — (TIF) [file pone.0265334.s006.tif]

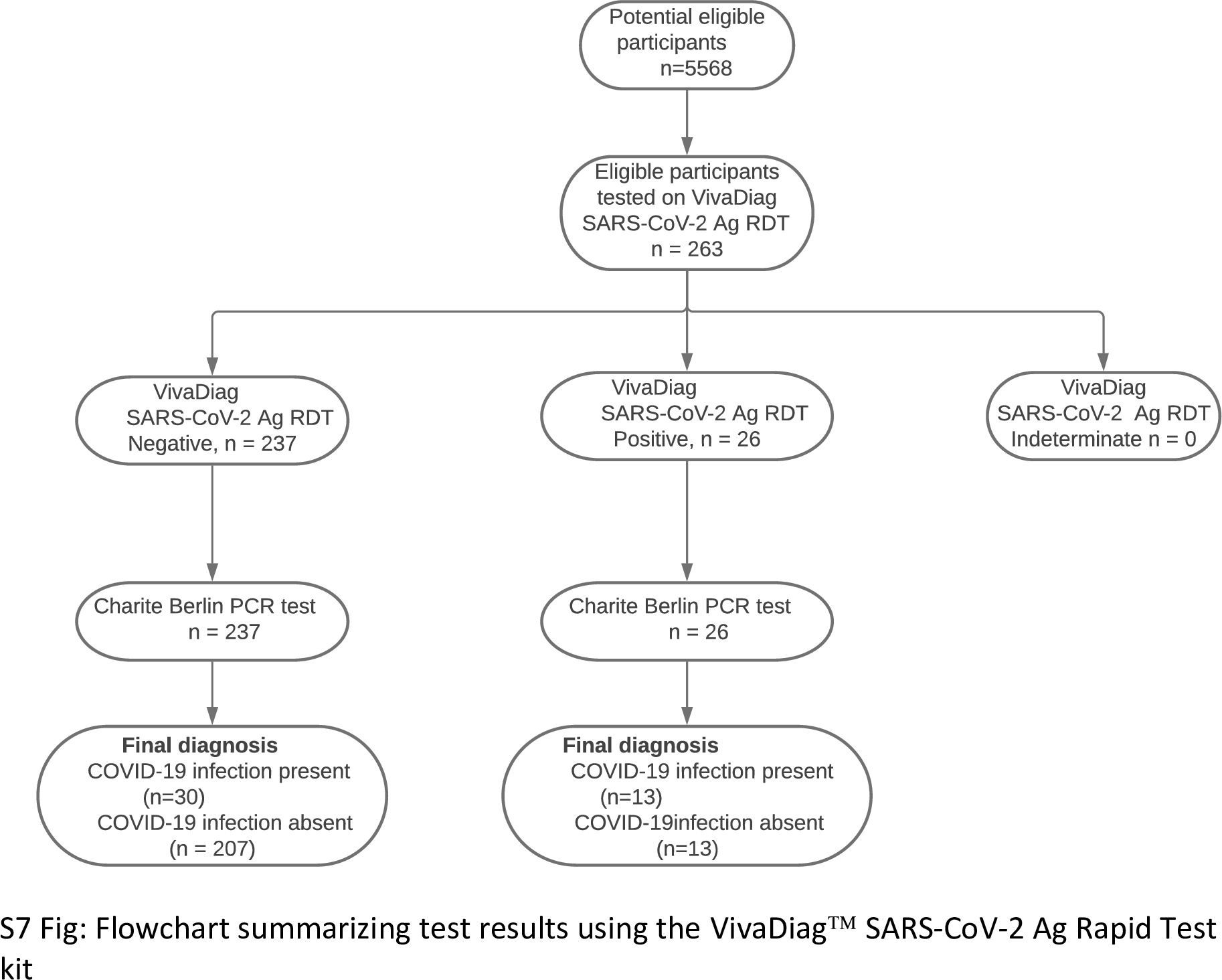

Supplement: S7 Fig — (TIF) [file pone.0265334.s007.tif]
