# Supplementary material for: Field evaluation of the performance of seven Antigen Rapid diagnostic tests for the diagnosis of SARs-CoV-2 virus infection in Uganda
Source: PLoS One. 2022 May 10;17(5):e0265334. doi: 10.1371/journal.pone.0265334 (PMC9089886; doi:10.1371/journal.pone.0265334)
Supplement: S1 Table — (DOCX) [file pone.0265334.s008.docx]

**S1 Table: Principal and Kit performance of the seven evaluated COVID -19 Rapid Antigen test kits**

| **Kit** | **Principle** | **Manufacturer’s Sensitivity (%)** | **Manufacturer’s Specificity (%)** | **Reference** |
| --- | --- | --- | --- | --- |
| BIOCREDIT COVID -19 Ag | Colloid gold conjugate pad and a membrane strip pre-coated with antibodies specific to SARS-CoV-2 antigen. When SARS-CoV-2 antigen is present, a visible black band appears on the test line as antibody-antigen-antibody gold conjugate complex forms | 92.0 | 98.0 | https://www.biovendor.com/biocredit-covid-19-ag-detection-kit?utm_source=google&utm_medium=organic |
| COVID-19 Ag Respi-Strip | Nitrocellulose membrane sensitised with monoclonal antibodies against SARS-CoV and SARS-CoV-2 highly conserved nucleoprotein antigen. Another monoclonal antibody is conjugated to colloidal gold nanoparticles and immobilised on the membrane. If SARS-CoV-2 antigen is present in the sample, the conjugate-SARS-CoV-2 complex will remain bound to the anti-SARS-CoV-2 antibody immobilized on the nitrocellulose | 1. 60 2. 60.3 | 1. 100 2. 98.3 | https://www.corisbio.com/products/covid19.php |
| PCL COVID19 Ag Rapid FIA | COVID19 antibody is immobilised in the test region of the nitrocellulose membrane. When SARS-CoV-2 antigen is present, it binds to the conjugate and is moved by capillary principle and is immobilised with the antibody in the test region. Antigen-antibody immune responses form double complexes that fluoresce. | 100?? | 97.8 |  |
| MEDsan® SARS-CoV-2 Antigen Rapid test | The conjugate pad contains colloidal gold conjugated to SARS-CoV-2 antibodies (test line) and mouse IgG-gold conjugates (control line). When SARS-CoV-2 antigen is present, it binds to the SARS-CoV-2 conjugates forming an antigen antibody complex | 92.5  (96.5 % for samples with Ct values ≤33) | 99.8 | https://www.medsan.eu/en/medsan-sars-cov-2-antigen-rapid-test-eu-version/ |
| Panbio™ COVID-19 Ag Rapid test | Membrane strip is pre-coated with immobilized SARS-CoV-2 antibody (test line) and mouse monoclonal anti-chicken IgY (control line). Two types of conjugates (human IgG specific to SARS-CoV-2 Ag gold conjugate and chicken IgY gold conjugate move on the membrane chromatographically and react with anti-SARS-CoV-2 antibody and pre-coated anti-chicken IgY respectively. | 91.4 | 99.8 | <https://www.globalpointofcare.abbott/en/product-details/panbio-covid-19-ag-antigen-test.html> |
| Novegent COVID-19 Antigen Rapid test kit (colloidal gold) | A colloidal gold conjugated mouse monoclonal antibody that specifically binds to the COVID-19 virus antigen (and is used as a tracer for the virus nucleoprotein) and a mouse monoclonal antibody against the nucleoprotein are immobilised onto the nitrocellulose membrane. A specimen positive for SARS-CoV-2 produces a purple red test line formed by the specific antigen-colloidal gold conjugated antibody-immobilized antibody complex | 98.3 | 99.2 | <http://www.novegent.eu/antigen/> |
| VivaDiag™ SARS-CoV-2 Ag Rapid Test kit | Test is based on the immunoassay technology. Anti-SARS coronavirus monoclonal antibody is coated on the test line and anti-mouse IgG polyclonal antibody on the control line. Presence of SARS-CoV-2 antigen gives a purplish-red test line | 82.86 | 100 | <https://www.vivachek.com/en/prods/sarscov2-ag-rapidtest.html> |
