# Supplementary material for: Field evaluation of the performance of seven Antigen Rapid diagnostic tests for the diagnosis of SARs-CoV-2 virus infection in Uganda
Source: PLoS One. 2022 May 10;17(5):e0265334. doi: 10.1371/journal.pone.0265334 (PMC9089886; doi:10.1371/journal.pone.0265334)
Supplement: Attachment — Submitted filename: Reviewer Recommendation and Comments for Manuscript Number PONE.docx [file pone.0265334.s009.docx]

**Reviewer Recommendation and Comments for Manuscript Number PONE-D-21-24051**

The comparison of the panel of tests is key to improving the use of these tests in local situation. Such is very important and carrying out such investigations improve the performance as mitigation measures can be put in place in real time. The fact that such tests can be performed at the bedside therefore hasten the recovery process and facilitates a speedy intervention and therefore recovery.

**Observation**

1. There is need for the authors to revisit the figures (number/values) in the text as well as those in the tables in order to align them e.g. the Table 1 says 245 (instead of 249) and Table 2 says 66* while the text in Line No. 220 is referring to sixty seven.

2. The presentation of the Table is little complicated as it is not clarified as to what is presented in the Table and what is not. In would be helpful for the reader to cite the Table or Figure at the first mention. The way the results are presented, the reader will only know where the table being referred to is when they reach the end of the paragraph.

3. In addition, navigating through the text and tables is a little tedious as the data is not presented according to the tables order. In the process, making locating what is being referred to in the text a little hard for the reader. For instance, Lines 192-197 appears to be referring to both Table 1 (asymptomatic participants) and Table 2(sensitivity). It would also be helpful for the reader if the authors can state when the results are "not shown" or are found in the supplementary.

4. The presentation of the results can be made clear by removing duplicate text in the row or columns e.g. Test and CT Value can be condensed in order to improve clarity of the results.

5. Demoting or promoting the subheading of the "test" and "performance" for each test would remove extra texts and improve legibility and clutter regarding results presentation. If allowable, assigning pseudo names such as Biocredit, Respi-strip, PCL COVID, MEDsan, Panbio, Novegent, VivaDiag could make reading easier unlike using the full name of the test at every mention of the test.

6. Beside the recommended performance by WHO, the authors i was unable to determine what other criteria was employed to arrive at the recommendation of the 2 rapid tests, although statistical analysis was mentioned. It would be interesting to know how the tests compare to each other or how one test can complement the other based on the inherent characteristics of the test. Do these other tests that were not recommended have any superior characteristics? compared to the other two?

7. Are there any factors that the authors could speculate that could have caused the deviation of the test from the manufacturer’s recommendations? Are the steps that can be taken to salvage the performance of the other tests?
