# Supplementary material for: Field evaluation of the performance of seven Antigen Rapid diagnostic tests for the diagnosis of SARs-CoV-2 virus infection in Uganda
Source: PLoS One. 2022 May 10;17(5):e0265334. doi: 10.1371/journal.pone.0265334 (PMC9089886; doi:10.1371/journal.pone.0265334)
Supplement: Attachment — Submitted filename: Response to Reviewers.docx [file pone.0265334.s010.docx]

**Response to Reviewers**

PONE-D-21-24051

Field Evaluation of the Performance of Seven Antigen Rapid Diagnostic Tests for the Diagnosis of SARs-CoV-2 Virus Infection in Uganda

1. Information in the tables must be well presented and numbers must tally with those that are given in the text. The order of presentation of results in the text must follow the order in which they appear in the tables.

**Response:**

The information in the tables and numbers in text have been reviewed and now tally. The order of the results in the text has been reorganised to follow the order in which they appear in the tables.

2. Provide more information about the participants in this study and how they were selected. Do they represent the population on which such the tests are intended to be used on? Was each test applied to every participant and if not, what impact would this have on the comparison of the test results.

**Response**

The participants were enrolled consecutively whenever RDT kits for evaluation were supplied to UVRI. The evaluation did not receive the RDTs at the same time and quantities supplied for the evaluation were different (depended on how many the suppliers would be able to supply). The majority of participants considered for the evaluation were those found admitted with symptoms known to be for COVID-19 patients. Since RDTs were not received at the same time and because of the urgency due to the pandemic, we were not able to evaluate the kits using the same participants as we would have wanted but rather to enrol those who consented to the evaluation. This is further complicated by the acute nature of the disease and rapid changes in viral loads in individuals. We observed that our sample had significantly more men than women and this was also observed in the centres used (more men in health facilities and border crossings). This means that if the RDTs are to be used at health facilities where individuals with COVID-19 like symptoms would go for health care, or at border crossings, then it is more likely that a similar profile of individuals will be the ones to be tested using recommended RDTs.

3. Provide more information about the validity of the RT-PCR that was used as a Gold Standard in this study.

**Response**

More information on the sensitivity and specificity of the RT-PCR that was used as the Gold Standard in this study has been included in the manuscript.

Line162-166: The protocol has good sensitivity with a limit of detection (LoD) of 3.9 RNA copies per reaction for E-gene assay and 3.6 RNA copies per reaction for RdRp gene assay using in-vitro transcribed RNA identical to 2019 novel coronavirus sequences and specificity of 100%

If applicable, we recommend that you deposit your laboratory protocols in [protocols.io](http://protocols.io/) to enhance the reproducibility of your results. Protocols.io assigns your protocol its own identifier (DOI) so that it can be cited independently in the future. For instructions see: <https://journals.plos.org/plosone/s/submission-guidelines#loc-laboratory-protocols>. Additionally, PLOS ONE offers an option for publishing peer-reviewed Lab Protocol articles, which describe protocols hosted on [protocols.io](http://protocols.io/). Read more information on sharing protocols at <https://plos.org/protocols?utm_medium=editorial-email&utm_source=authorletters&utm_campaign=protocols>.

**Response**

 Protocol used has been referenced. Not deposited in the protocols.io .

When submitting your revision, we need you to address these additional requirements.

1. Please ensure that your manuscript meets PLOS ONE's style requirements, including those for file naming. The PLOS ONE style templates can be found at
   <https://journals.plos.org/plosone/s/file?id=wjVg/PLOSOne_formatting_sample_main_body.pdf> and 
   <https://journals.plos.org/plosone/s/file?id=ba62/PLOSOne_formatting_sample_title_authors_affiliations.pdf>

**Response:**

Plos One formatting requirements have been addressed

2.Please include additional information regarding the survey or questionnaire used in the study and ensure that you have provided sufficient details that others could replicate the analyses. For instance, if you developed a questionnaire as part of this study and it is not under a copyright more restrictive than CC-BY, please include a copy, in both the original language and English, as Supporting Information.

**Response:**

No questionnaire has been included. Because the survey form was in excel. A copy of the Excel dataset used for data collection and which was transformed to STATA for the analysis can be accessed using the link below;-

<http://eaccr.org/sites/default/files/2021-11/Field%20Evaluation%20of%20the%20Performance%20of%20Seven%20Antigen%20Rapid%20Diagnostic%20Tests.xlsx>

3. Thank you for stating the following in the Competing Interests section: 
[RD is a consultant at Abbott. Therefore, he did not participate in the investigation of Panbio™ COVID-19 Ag Rapid test performance.].

Please confirm that this does not alter your adherence to all PLOS ONE policies on sharing data and materials, by including the following statement: "This does not alter our adherence to  PLOS ONE policies on sharing data and materials.” (as detailed online in our guide for authors <http://journals.plos.org/plosone/s/competing-interests>).  If there are restrictions on sharing of data and/or materials, please state these. Please note that we cannot proceed with consideration of your article until this information has been declared.

Please include your updated Competing Interests statement in your cover letter; we will change the online submission form on your behalf.

**Response**

**Updated conflict of interest statement:**

RD is a consultant at Abbott. Therefore he did not participate in the investigation of Panbio™ COVID-19 Ag Rapid test performance. This does not alter our adherence to PLOS ONE policies on sharing data and materials.

4. In your Data Availability statement, you have not specified where the minimal data set underlying the results described in your manuscript can be found. PLOS defines a study's minimal data set as the underlying data used to reach the conclusions drawn in the manuscript and any additional data required to replicate the reported study findings in their entirety. All PLOS journals require that the minimal data set be made fully available. For more information about our data policy, please see <http://journals.plos.org/plosone/s/data-availability>.

Upon re-submitting your revised manuscript, please upload your study’s minimal underlying data set as either Supporting Information files or to a stable, public repository and include the relevant URLs, DOIs, or accession numbers within your revised cover letter. For a list of acceptable repositories, please see <http://journals.plos.org/plosone/s/data-availability#loc-recommended-repositories>. Any potentially identifying patient information must be fully anonymized.

Important: If there are ethical or legal restrictions to sharing your data publicly, please explain these restrictions in detail. Please see our guidelines for more information on what we consider unacceptable restrictions to publicly sharing data: <http://journals.plos.org/plosone/s/data-availability#loc-unacceptable-data-access-restrictions>. Note that it is not acceptable for the authors to be the sole named individuals responsible for ensuring data access.

We will update your Data Availability statement to reflect the information you provide in your cover letter.

**Response:** The minimum Dataset has been made available. It can be accessed using the link below;-

<http://eaccr.org/sites/default/files/2021-11/Field%20Evaluation%20of%20the%20Performance%20of%20Seven%20Antigen%20Rapid%20Diagnostic%20Tests.xlsx>

5. One of the noted authors is a group or consortium [EPI Laboratory team and UVRI COVID 19 Technical team.] In addition to naming the author group, please list the individual authors and affiliations within this group in the acknowledgments section of your manuscript. Please also indicate clearly a lead author for this group along with a contact email address.

**Response:**

Line 651-670: The members in EPI Laboratory team and UVRI COVID 19 Technical team have been named in the acknowledgement section. The author affiliation has been included and the Lead authors in the group named.

6. We note that you have included the phrase “data not shown” in your manuscript. Unfortunately, this does not meet our data sharing requirements. PLOS does not permit references to inaccessible data. We require that authors provide all relevant data within the paper, Supporting Information files, or in an acceptable, public repository. Please add a citation to support this phrase or upload the data that corresponds with these findings to a stable repository (such as Figshare or Dryad) and provide and URLs, DOIs, or accession numbers that may be used to access these data. Or, if the data are not a core part of the research being presented in your study, we ask that you remove the phrase that refers to these data.

**Response:** The statement with this reference has been deleted from the manuscript. It was not a core part of the research being presented.

[Note: HTML markup is below. Please do not edit.]

Reviewers' comments:

Reviewer's Responses to Questions

**Comments to the Author**

1. Is the manuscript technically sound, and do the data support the conclusions?

The manuscript must describe a technically sound piece of scientific research with data that supports the conclusions. Experiments must have been conducted rigorously, with appropriate controls, replication, and sample sizes. The conclusions must be drawn appropriately based on the data presented.

Reviewer #1: Partly

Reviewer #2: Yes

Reviewer #3: Partly

2. Has the statistical analysis been performed appropriately and rigorously?

Reviewer #1: Yes

Reviewer #2: No

Reviewer #3: Yes

3. Have the authors made all data underlying the findings in their manuscript fully available?

The [PLOS Data policy](http://www.plosone.org/static/policies.action#sharing) requires authors to make all data underlying the findings described in their manuscript fully available without restriction, with rare exception (please refer to the Data Availability Statement in the manuscript PDF file). The data should be provided as part of the manuscript or its supporting information, or deposited to a public repository. For example, in addition to summary statistics, the data points behind means, medians and variance measures should be available. If there are restrictions on publicly sharing data—e.g. participant privacy or use of data from a third party—those must be specified.

Reviewer #1: No

Reviewer #2: Yes

Reviewer #3: No

**Response:** The data has been made available. It can be found using the following URL

<http://eaccr.org/sites/default/files/2021-11/Field%20Evaluation%20of%20the%20Performance%20of%20Seven%20Antigen%20Rapid%20Diagnostic%20Tests.xlsx>

4. Is the manuscript presented in an intelligible fashion and written in standard English?

PLOS ONE does not copyedit accepted manuscripts, so the language in submitted articles must be clear, correct, and unambiguous. Any typographical or grammatical errors should be corrected at revision, so please note any specific errors here.

Reviewer #1: Yes

Reviewer #2: Yes

Reviewer #3: Yes

5. Review Comments to the Author

Please use the space provided to explain your answers to the questions above. You may also include additional comments for the author, including concerns about dual publication, research ethics, or publication ethics. (Please upload your review as an attachment if it exceeds 20,000 characters)

Reviewer #1: The manuscript is interesting and well written.
The authors should acknowledge more limitations of the study. The comparison between the tests is not fair, because the tests are not compared in a randomized way. Sensitivity and specificity can be different if samples come from populations with different disease spectrum/prevalence.

The STARD checklist should be used (<https://www.equator-network.org/reporting-guidelines/stard>).

**Response:**

**Line 625-631 :** Limitations why we could not use the mentioned randomization design have been added in the manuscript. Basically these kits were evaluated as and when the suppliers brought them to the UVRI. This was at different times and in different quantities. Because of the urgency to identify an RDT that would help in the diagnosis of COVID-19 we evaluated as soon as we received the kits and used participant sources established by the Ugandan Ministry of Health (Isolation centres and points of entry)

-We have followed the STARD checklist when reporting in the manuscript.

Table 1 should be completed with percentages for all the variables (e.g. age groups).

**Response**

Table has been completed with percentages for all the variables (age groups and the grouping according to duration of symptoms now have percentages).

Tables should be formatted in a better way.

**Responses**

Table 1, 2 and 3 have been formatted.

Is the time lag between Antigen Test and PCR a possible explanation for the difference observed with vendors diagnostic performance?

**Response:**

There is a time lag between the Antigen test and PCR which range from 1 to 37 days with a median of 4-7 days except for RESPI Strip which had a median of 21 days. We think this did not affect the diagnostic performance of the kits since the samples were stored at -80^o^C during that time. Please see table below: This information has been included in the manuscript.

**Table : Days between the Rapid Diagnostic Test and the PCR test**

| **Ag RDT** | **Median days** | **Mean** | **Min** | **Max** |
| --- | --- | --- | --- | --- |
| Biocredit | 4 | 7.3 | 1 | 21 |
| Respi | 21 | 22.7 | 1 | 37 |
| PCL | 7 | 9.7 | 1 | 24 |
| MedSan | 5 | 4.9 | 2 | 12 |
| PanBio | 4 | 3.7 | 2 | 7 |
| Colloidal/Novegent | 4 | 4.3 | 1 | 11 |
| VivaDiag | 5 | 7.5 | 2 | 28 |

**Reviewer #2: Reviewer Recommendation and Comments for Manuscript Number PONE-D-21-24051**
The comparison of the panel of tests is key to improving the use of these tests in local situation. Such is very important and carrying out such investigations improve the performance as mitigation measures can be put in place in real time. The fact that such tests can be performed at the bedside therefore hasten the recovery process and facilitates a speedy intervention and therefore recovery.
Observation
1. There is need for the authors to revisit the figures (number/values) in the text as well as those in the tables in order to align them e.g. the Table 1 says 245 (instead of 249) and Table 2 says 66* while the text in Line No. 220 is referring to sixty seven.

**Response:** We have aligned the figures in the tables and text.

2. The presentation of the Table is little complicated as it is not clarified as to what is presented in the Table and what is not. In would be helpful for the reader to cite the Table or Figure at the first mention. The way the results are presented, the reader will only know where the table being referred to is when they reach the end of the paragraph.

**Response**

We have maintained reference to the table at the end since we did not report per table but per kit. Referring to the table at the beginning for each kit would make the presentation monotonous

3. In addition, navigating through the text and tables is a little tedious as the data is not presented according to the tables order. In the process, making locating what is being referred to in the text a little hard for the reader. For instance, Lines 192-197 appears to be referring to both Table 1 (asymptomatic participants) and Table 2(sensitivity). It would also be helpful for the reader if the authors can state when the results are "not shown" or are found in the supplementary.

**Response**

We have rearranged the text to follow the order of the tables presented for the specific kit being evaluated.

**Reviewer #3**: This is a nice evaluation of seven RDTs for covid. However, diagnostic accuracy depends a lot on the study sample and participant mix. This is information that is lacking in this study and it is what I have most concerns about.

1. Participants were selected from travellers and people in isolation. To what extent are they representative for the population in which the RDTs will be used? For example, the conclusion says: "This RDT can be recommended for use among symptomatic patients in health facilities in order not to delay patient management while waiting for qRT-PCR results". But these are not the participants included.

**Response**

The participants were enrolled consecutively whenever RDT kits for evaluation were supplied to UVRI. The evaluation did not receive the RDTs at the same time and quantities supplied for the evaluation were different (depended on how many the suppliers would be able to supply). The majority of participants considered for the evaluation were those found admitted with symptoms known to be for COVID-19 patients. Since RDTs were not received at the same time and because of the urgency due to the pandemic, we were not able to evaluate the kits using the same participants as we would have wanted but rather to enrol those who consented to the evaluation. This is further complicated by the acute nature of the disease and rapid changes in viral loads in individuals. We observed that our sample had significantly more men than women and this was also observed in the centres used (more men in health facilities and border crossings). This means that if the RDTs are to be used at health facilities where individuals with COVID-19 like symptoms would go for health care, or at border crossings, then it is more likely that a similar profile of individuals will be the ones to be tested using recommended RDTs.

In addition, the Isolation Centers had patients that were admitted from different parts of the country.

2. For the reason mentioned in the previous comment, I do not think that this conclusion is valid. So please rephrase.

**Response**

Line 593-604: The conclusion has been rephrased.

3. How were particapants selected? In a consecutive way? On particular days? This needs to be clarified.

**Response**

Participants were recruited consecutively, and these were found at the sites of recruitment. Also recruitment for the different kits was as when the kits were received from the suppliers.

We have clarified this in the manuscript.

4. How were the different tests allocated to the right person? At random, or in different time-periods? Please explain.

**Response**

The different kits were allocated at different time periods as the different tests were received from the suppliers.

The dates when the different participants for the different kits were recruited is mentioned in the manuscript

5. How many samples/tests per participant were included? Did some participants receive more than two tests? Please explain.

Most participants/samples had only one test. This was because of how we received the samples and also when samples were taken off, they were put in the buffer supplied with that kit. A few samples were used to evaluate more than one test however a comparison of the results has not been included in the manuscript. Additionally, one RDT (RESPI) did not have a buffer but shared a sample with real time PCR.

Seventy- six samples had both PCL COVID19 Ag Rapid FIA and COVID-19 Ag Respi-Strip. Fifteen samples had both BIOCREDIT COVID -19 Ag Test and COVID-19 Ag Respi-Strip used. As mentioned above, no comparison between results from these tests has been included in the manuscript.

Line 332-335: The information about numbers of samples used to evaluate more than one RDT has been included in the manuscript.

6. There are distinct differences in population mix (males/females; cases/controls) between the different tests. This could have influenced the accuracy, but the authors say little about these differences. Maybe this can be added?

**Response**

The population mix (cases/controls ) between the different tests has been added as a study limitation.

However, we do not think the difference in male/female mix could have affected the accuracy of the different tests.

**Response**

7. In general, I think this manuscript would benefit from a better reporting. Please refer to the STARD guidelines for reporting of diagnostic accuracy studies.

**Response**

We have referred to the STARD guidelines for reporting diagnostic accuracy studies and have made some edits to the manuscripts.

8. RT-PCR is the reference standard, but very little information is provided. For example, I can imagine that having only one negative RT-PCR is not very reliable in determining a 'control' participant. Would it be possible to explian a bit more about the reference standard? And could the reference standard have led to bias? (which should then be mentioned in a limitations section in the Discussion)

**Response**

Line 162-166: We have added more information concerning the RT-PCR we used as the reference standard: This assay was selected as the Standard reference for the kit evaluation because the protocol has good sensitivity with a limit of detection (LoD) of 3.9 RNA copies per reaction for E-gene assay and 3.6 RNA copies per reaction for RdRp gene assay using invitro transcribed RNA identical to 2019 novel coronavirus sequences and specificity of 100%. It was also recommended by WHO.

Since the specificity of the test was 100%, we believe using only one negative RT-PCR result for the control participants did not cause any bias in our study.

9. The authors have analyzed the data against different Ct thresholds. As far as I can see, the Ct thresholds are different from what I have seen in other RDT analyses. Would it be possible for the authors to comment on these Ct thresholds? And how do they relate to clinical practice? Why were those Ct's chosen?

**Response**

The authors chose the Ct-thresholds in reference to literature and after discussions in-country.

A Ct ≤ 39: This is the cut off for positivity provided by the Charite Berlin PCR Protocol that we used in the analysis (Corman VM et al., 2020).

A Ct≤29 was used because literature showed that patients with Ct value below 30 were found to be more infectious due to the high viral load and these Ct values were more likely to be found in the first days of the infection. Since we are looking for kits that will detect the infection early we decided to include this cut off in our analysis.

[*https://www.sciencemag.org/news/2020/09/one-number-could-help-reveal-how-infectious-covid-19-patient-should-test-results*](https://www.sciencemag.org/news/2020/09/one-number-could-help-reveal-how-infectious-covid-19-patient-should-test-results)

*(Bayat SA et al. 2021)*

A Ct value of ≤ 33 for the E-gene were found not to be contagious. No viruses were cultured from the patients with Ct value greater than 34 in a study carried out in France. Therefore, these were taken to be of less clinical value (La Scola B et al. 2020). Since we still need to identify these patients that is why we had this cut off value although our final report is on RdRp gene Ct value.

6. PLOS authors have the option to publish the peer review history of their article ([what does this mean?](https://journals.plos.org/plosone/s/editorial-and-peer-review-process#loc-peer-review-history)). If published, this will include your full peer review and any attached files.


If you choose “no”, your identity will remain anonymous but your review may still be made public.


**Do you want your identity to be public for this peer review?** For information about this choice, including consent withdrawal, please see our [Privacy Policy](https://www.plos.org/privacy-policy).

Reviewer #1: No

Reviewer #2: **Yes:** Ngonda Saasa

Reviewer #3: **Yes:** Mariska Leeflang
