# Supplementary material for: Field evaluation of the performance of seven Antigen Rapid diagnostic tests for the diagnosis of SARs-CoV-2 virus infection in Uganda
Source: PLoS One. 2022 May 10;17(5):e0265334. doi: 10.1371/journal.pone.0265334 (PMC9089886; doi:10.1371/journal.pone.0265334)
Supplement: Attachment — Submitted filename: Responses to Reviewers.docx [file pone.0265334.s011.docx]

**Responses to Reviewers**

PONE-D-21-24051R1

Field Evaluation of the Performance of Seven Antigen Rapid Diagnostic Tests for the Diagnosis of SARs-CoV-2 Virus Infection in Uganda

1. Please review your reference list to ensure that it is complete and correct. If you have cited papers that have been retracted, please include the rationale for doing so in the manuscript text, or remove these references and replace them with relevant current references. Any changes to the reference list should be mentioned in the rebuttal letter that accompanies your revised manuscript. If you need to cite a retracted article, indicate the article’s retracted status in the References list and also include a citation and full reference for the retraction notice.

**Response:**

References have been reviewed and changes have been made to reference No 6 and 7.

**Reference no 6**. Bulilete O, Lorente P, Leiva A, Carandell E, Oliver A, Rojo E, et al. Evaluation of the Panbio rapid antigen test for SARS-CoV-2 in primary health care centers and test sites. medRxiv. 2020.

Replaced with more up to date reference below:

Bulilete O, Lorente P, Leiva A, Carandell E, Oliver A, Rojo E, Pericas P, Llobera J, COVID-19 Primary Care Research Group. Panbio™ rapid antigen test for SARS-CoV-2 has acceptable accuracy in symptomatic patients in primary health care. Journal of Infection. 2021 Mar 1;82(3):391-8.

**Reference no 7**.Olearo F, Noerz D, Heinrich F, Sutter JP, Roedel K, Schultze A, et al. Handling and accuracy of four rapid antigen tests for the diagnosis of SARS-CoV-2 compared to RT-qPCR. medRxiv. 2020.

Replaced with the reference below: The reference above is no longer on the website. The reference available is the one below

Olearo F, Nörz D, Heinrich F, Sutter JP, Roedl K, Schultze A, Zur Wiesch JS, Braun P, Oestereich L, Kreuels B, Wichmann D. Handling and accuracy of four rapid antigen tests for the diagnosis of SARS-CoV-2 compared to RT-qPCR. Journal of Clinical Virology. 2021 Apr 1;137:104782.

2. Thanks for having addressed all my comments. I think that the manuscript is improved but there is still one thing missed. STARD checklist should be uploaded as supplementary material and the authors should explicitly state in what page/section the checklist item has been covered.

**Response**

The STARD checklist has been attached to the rebuttal letter. In the checklist we have indicated the line no where the checklist has been covered. We have not included the STARD checklist in the supplementary material since we could not reference it in the manuscript

3. The selection criteria have not been clearly described. The authors now describe that participants were enrolled consecutively, but it is not clear in what setting the study was done or whether the patients enrolled in this study were representative. Also, the authors write that they included cases and controls, but that is ambiguous language. Please check the STARD guidelines about what to report about the selection criteria and the selection process.

**Response**

**Line 84-93**. More information has been included in the section of the selection criteria and process.

**Line 27-28**. Cases and Controls have been replaced with PCR positive and PCR negative participants.

4. The authors state that they followed the STARD guidelines, but there is still information missing. Please check. Also, the STARD guidelines recommend a flow chart to describe visually how patients enroll in the study and the tests they undergo and what the test results are. Such a flow chart would be really helpful in this case.

**Response**

- We have reviewed the STARD guidelines to guide us on inclusion of missing information in the manuscript. The STARD checklist has been attached to indicate where the different sections of the STARD checklist are in the paper.
- Flow charts showing patient enrollment and tests have been placed as figures in the supplementary material.

5. From the response to my comment about the timing of the tests, I understand that every patient is tested by only one of the index tests (and the reference standard). So patients enrolled at the start of the study will have received a different test than patients enrolled at the end of the study. That makes a comparisons between tests a bit difficult. So actually, authors should have mentioned this as a limitation. (see also my previous comment 6)

**Response**

**Line 491-493.** “Difficult in comparison of the tests because of timing of the tests and thus every patient tested with one of the index tests” has been included in the study limitation.

6. The authors provided now more information about the reference standard, but not how it was really used to make a diagnosis (e.g. for a participant who does not have COVID-19, should the test have been negative twice?).

**Response : Line 137-146:**

This has now been described**:**  A sample was considered positive if it had a Ct value ≤39 on the confirmatory qRT-PCR. A sample was considered negative if the Ct value was undetermined on the screening qRT-PCR or if it was positive on the screening qRT-PCR but undetermined or >39 on the confirmatory qRT-PCR.
